# Hematologic Markers as Prognostic Factors in Nonmetastatic Esophageal Cancer Patients under Concurrent Chemoradiotherapy

**DOI:** 10.1155/2019/1263050

**Published:** 2019-01-29

**Authors:** Yi-Feng Wu, Sung-Chao Chu, Bee-Song Chang, Yi-Tso Cheng, Tso-Fu Wang

**Affiliations:** ^1^Department of Hematology and Oncology, Buddhist Tzu Chi General Hospital, Hualien, Taiwan; ^2^Department of Oncology, Tzu-Chi University, Hualien, Taiwan; ^3^Department of Cardiovascular Surgery, Buddhist Tzu Chi General Hospital, Hualien, Taiwan

## Abstract

Nonmetastatic esophageal cancer can demonstrate a high local recurrence rate even under the standard treatment. We evaluated platelet counts before and after concurrent chemoradiotherapy (CCRT), neutrophil-to-lymphocyte ratio, and platelet-to-lymphocyte ratio for predicting esophageal cancer prognosis under CCRT. Newly diagnosed patients with esophageal cancer (stages IA–IIIC) who underwent CCRT during January 2013–December 2017 were enrolled. The data were collected retrospectively. Overall survival (OS), time to progressive disease (TPD), and time to metastasis (TM) were recorded for indicating prognosis. Kaplan–Meier curves were plotted and univariate and multivariate analyses were performed. In total, 105 patients were enrolled. The stages of esophageal cancer and surgery were associated with prognosis (i.e., OS, TPD, and TM). Based on TPD and TM, women had better prognosis than men. In the univariate analysis, high pre- and post-CCRT platelet counts (>300,000/*μ*L), platelet-to-lymphocyte ratio (PLR), and neutrophil-to-lymphocyte ratio (NLR) as well as low lymphocyte percentage were significantly associated with poor prognosis. However, in the multivariate analysis, only post-CCRT high platelet count (>300,000/*μ*L) remained significantly associated with poor prognosis (P = .041, .045, and .023 for OS, TPD, and TM, respectively). Poor prognosis was observed in patients with high platelet counts, PLR, NLR, and low lymphocyte percentage. Surgery was an independent factor predicting better prognosis. Our findings may have clinical significance with regard to therapeutic decision-making.

## 1. Introduction

Esophageal cancer is the eighth most common cancer worldwide and the sixth most common cause of cancer-related death [1]. In Taiwan, it was the ninth most common cancer in 2016, with a mortality rate of 4.8 per 100,000 people. In Hualien, Taiwan, the mortality rate is 7.3 per 100,000 people. Most esophageal cancers (more than 90%) in Taiwan are squamous cell carcinomas [2]. Trimodality therapy comprising neoadjuvant concurrent chemoradiotherapy (CCRT) followed by esophagectomy has become the treatment standard for locally advanced esophageal cancer [3, 4]. However, only 30%–40% of the patients can undergo surgery [5]. The standard nonsurgical treatment option is mainly based on the results of the Radiation Therapy Oncology Group (RTOG) 85-01 study, which showed that definite CCRT had a 10-year survival rate of 20% only [6, 7]. However, a high local recurrence rate of 46% was reported after definite CCRT in the RTOG and RTOG trials [8]. Current staging system might not predict outcome with the same stage, and lack of efficiently prognostic biomarkers is responsible for the high mortality rates caused by esophageal cancer.

The relationship between cancer and thrombosis was established in the late 19th century by Armand Trousseau. Since then, thrombocytosis has been associated with cancer prognosis. Clinical studies have investigated the frequency of high platelet count in patients with cancer and the role of high platelet count in patient outcomes. The overall survival (OS) of patients with ovary cancer [9], lung cancer [10], and breast cancer [11] has been related to thrombocytosis at the time of diagnosis. Except diagnosis, poor prognosis of colorectal cancer [12] and renal cancer [13] are related to high platelet counts at presurgery. Sylman et al. reported that platelet count is a predictor of metastasis and venous thromboembolism in patients with cancer [14].

Systemic inflammation also plays a crucial role in cancer prognosis [15]. Inflammatory mediators are involved in apoptosis, angiogenesis, and DNA damage [16]. Numerous studies have confirmed the relationship between inflammation and cancer prognosis [17, 18]. Neutrophil-to-lymphocyte ratio (NLR) and platelet-to-lymphocyte ratio (PLR) are 2 common markers for cancer. For instance, a relatively high NLR or PLR has been reported to predict short progression-free survival and OS in many solid cancers [19–22].

Although numerous hematological markers have been identified and applied for predicting outcomes in patients of solid malignancies, for examples, thrombocytosis, NLR, and PLR, no consensus about above markers is associated with esophageal cancer prognosis exists. Moreover, no study has reported a relationship in platelet counts after CCRT for esophageal cancer. In this study, we evaluated platelet counts at pre- and post-CCRT, NLR, and PLR for predicting nonmetastatic esophageal cancer prognosis under CCRT.

## 2. Materials and Methods

### 2.1. Patients

Newly diagnosed patients with esophageal cancer (stages IA–IIIC) who received aggressive treatment with CCRT between January 2013 and December 2017 in Buddhist Tzu Chi General Hospital in Hualien were enrolled. Retrospective information included age, sex, and results of routine blood tests 1 week before the first and after the last radiotherapy or chemotherapy cycle. In the routine blood tests, white blood cell count, hemoglobin, platelet count, mean platelet volume, and differential white blood cell count were examined. A chemotherapy regimen with either cisplatin or carboplatin combined with continuous infusion of 5-fluorouracil was adopted. The patients undergoing only radiotherapy, oral chemotherapy, or supportive care were excluded. All patients had histologically confirmed squamous cell carcinoma. The stage was recorded through fluorodeoxyglucose positron emission tomography (FDG-PET) and tumor, node, and metastases (TNM) staging based on the American Joint Committee on Cancer's Cancer Staging Manual [23]. All patients received FDG-PET or computed tomography (CT) at least once after CCRT to examine the treatment response. This retrospective study was approved by the Institutional Review Board of Buddhist Tzu Chi General Hospital (IRB107-129-B).

### 2.2. Follow-Up Imaging

A follow-up imaging study was completed by each patient's physician. OS was defined based on time of death. The time to progressive disease (TPD) was defined as the duration from diagnosis to recurrence or metastasis according to the last imaging study and time to metastasis (TM) as the duration from diagnosis to metastasis according to the last imaging study.

### 2.3. Statistical Analyses

The collected data were entered into MedCalcR (version 9.6) for statistical analysis. OS, TPD, and TM were analyzed using Kaplan–Meier curves and the logrank test. The data are expressed as means and hazard ratios (HRs) and their 95% confidence intervals (CIs). Univariate and multivariate analyses adopted the Cox proportional-hazards regression. Univariate analysis variables with a* P* of <.05 were included in the multivariate analysis. A* P* of <.05 was considered to indicate statistical significance.

## 3. Results

### 3.1. Patient Characteristics

Overall, 105 patients were enrolled in this study from January 2013 to December 2017. The final follow-up date was July 30, 2018, and the mean follow-up duration was 586 days.  Table 1 lists patients' clinical pathological characteristics. The mean age at diagnosis was 57.69 (range, 38-81) years. Most patients were men (93.3%). Squamous cell carcinoma was confirmed for all patients through biopsy (100%). In total, 28 (26.7%), 48 (45.7%), and 29 (27.6%) patients had upper-, middle-, and lower-third tumors, respectively, and 13 (12.4%), 27 (25.7%), and 65 (61.9%) patients were at TNM stage I, II, and III, respectively. The laboratory data at diagnosis and after treatment are presented in Table 1. All patients underwent complete radiotherapy, and 96 (91.4%) of them finished CCRT with two-cycle chemotherapy.

After CCRT, 38 (36.2%), 48 (45.7%), 2 (1.9%), and 17 (16.2%) patients demonstrated complete remission, partial remission, stable disease, and progressive disease (PD), respectively. The overall response was 81.9%. After finishing CCRT, 38 (36.2%), 45 (42.9%), and 22 (20.9%) patients received esophagectomy, continuous chemotherapy without surgery, and palliative treatment alone, respectively. Until June 30, 2018, 56 patients (53.3%) had passed away. Overall, 62 patients (59%) with PD and 56 patients (53.3%) with metastasis were confirmed through FDG-PET, CT, or biopsy.

### 3.2. Patient Characteristics versus Esophageal Cancer Prognosis

As listed in Tables 2, 3, and 4, of the 105 patients in the univariate analysis, an advance stage of esophageal cancer was associated with poor prognosis for OS, TPD, and TM (*P* = .001, .008, and .004 respectively). Seven female patients had a better prognosis for TPD and TM (*P* = .014 and .027, respectively), but not for OS (*P* = .513). Overall, 38 (36.2%) patients underwent a surgery after CCRT, and compared with other patients, they demonstrated significantly better prognosis for OS, TPD, and TM (*P* = .013, .022, and .036, respectively), but not tumor site and age. The multivariate analysis results revealed that stage and surgery still demonstrated significant differences. For stage and surgery, the* P* values were respectively .013 for OS, .035 for TPD, and .040 for TM and .002 for OS, .005 for TPD, and .013 for TM. The doses of radiotherapy, days from diagnosis to initial treatment, and days from diagnosis to complete treatment were not associated with prognosis.

### 3.3. Platelet Count versus Esophageal Cancer Prognosis

**Figure 1:**
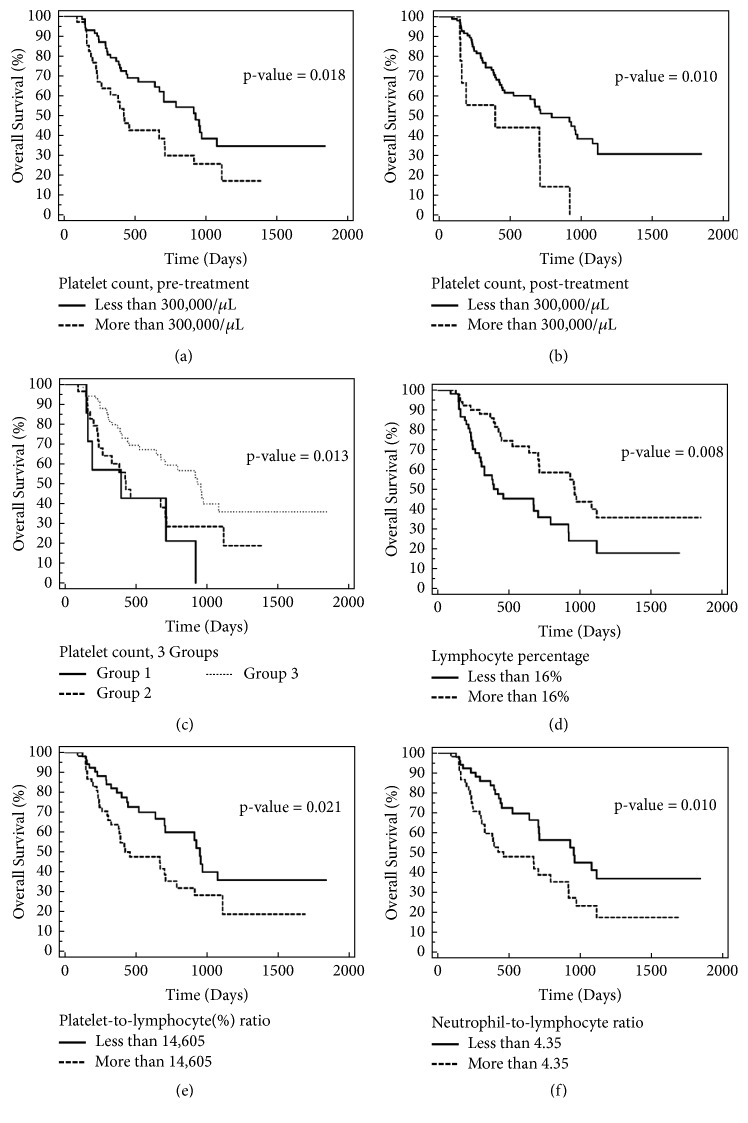
Overall survival curve based on platelet count at pretreatment, posttreatment, lymphocyte percentage, platelet-to-lymphocyte (%) ratio, and neutrophil-to-lymphocytes ratio (n=105). (a) Platelet count of more than 300,000/*μ*L at pretreatment was associated with poor prognosis. The median survival was 423 days in the high platelet count group and 928 days in the low platelet count group (*P* = .018). (b) Platelet count of more than 300,000/*μ*L at posttreatment was associated with poor prognosis. The median survival was 394 days in the high platelet count group and 791 days in the low platelet count group (*P* = .010). (c) Overall survival curve based on 3 groups. Group 1 was high platelet counts at pretreatment and posttreatment. Group 2 was a low platelet count at pretreatment with a high platelet count at posttreatment, or a high platelet count at pretreatment with a low platelet count at posttreatment. Group 3 was low platelet counts at pretreatment and posttreatment. The cutoff level for the platelet count was 300,000/*μ*L. The median OS for the groups 1, 2, and 3 was 394, 426, and 953 days, respectively (*P* = .013). (d) Low lymphocyte percentage (>16%) was associated with poor prognosis. The median survival was 423 days in the low group and 958 days in the high group (P = .008). (e) High platelet-to-lymphocyte (%) ratio (>14,605) was associated with poor prognosis. The median survival was 426 days in the high ratio group and 953 days in the low ratio group (*P* = .021). (f) High neutrophil-to-lymphocytes ratio (>4.35) was associated with poor prognosis. The median survival was 461 days in the high ratio group and 953 days in the low ratio group (*P* = .010).

High pre- and post-CCRT platelet counts (>300,000/*μ*L) were significantly associated with poor prognosis in the univariate analysis. At pretreatment and posttreatment,* P* values were, respectively, .018 for OS, .035 for TPD, and .012 for TM and .010 for OS, .042 for TPD, and .010 for TM. In the multivariate analysis, only posttreatment high platelet counts were significantly associated with poor prognosis (*P* = .041 for OS, .045 for TPD, and .023 for TM). The Kaplan–Meier curves (Figures 1(a) and 1(b)) revealed that, in the pretreatment and posttreatment groups, the median survival was, respectively, 423 and 394 days for platelet counts >300,000/*μ*L and 928 and 791 days for platelet counts <300,000/*μ*L.

We combined the data of the pretreatment and posttreatment platelet counts into 3 separate groups: group 1, high platelet counts at pretreatment and posttreatment; group 2, low platelet count at pretreatment with high platelet count at posttreatment or high platelet count at pretreatment with low platelet count at posttreatment; and group 3, low platelet counts at pretreatment and posttreatment. The cutoff level for the platelet count was 300,000/*μ*L. The univariate analysis revealed a significant association between platelet counts and OS (*P* = .013), but this association became nonsignificant in the multivariate analysis. The median OS in groups 1, 2, and 3 was 394, 426, and 953 days, respectively. The results showed that high platelet counts, at either pretreatment or posttreatment, were associated with poor prognosis (Figure 1(c)).

### 3.4. Lymphocyte Percentage, PLR, and NLR versus Esophageal Cancer Prognosis

With the median lymphocyte percentage cutoff set at 16%, a high lymphocyte percentage was associated with short OS, TPD, and TM in the univariate analysis (*P* = .008 for OS, .015 for TPD, and .021 for TM, Figure 1(d)). Platelet-to-absolute lymphocyte count (ALC) ratio and platelet-to-lymphocyte-percentage ratio were studied. The cutoff levels were established based on the median values: 236 and 14,605 for platelet-to-ALC and platelet-to-lymphocyte percentage ratios, respectively. Higher platelet-to-ALC and platelet-to-lymphocyte percentage ratios demonstrated poorer prognosis, except for platelet-to-ALC ratio for OS. For the platelet-to-ALC and platelet-to-lymphocyte percentage ratio,* P* values were, respectively, .083 for OS, .012 for TPD, and .003 for TM and .021 for OS, .007 for TPD, and .002 for TM. The NLR cutoff of the median (4.35) also demonstrated that a high level was significantly associated with poor prognosis (*P* = .010, .026, and .033 for OS, TPD, and TM, respectively). The aforementioned results were only the univariate analysis results (Tables 2, 3, and 4 and Figures 1(e) and 1(f)).

## 4. Discussion

Esophageal cancer is one of the most common cancers worldwide [1]. In Hualien, the mortality rate is 7.3 per 100,000 people. Studies on esophageal cancer often have included adenocarcinoma and squamous cell carcinoma, but squamous cell carcinoma accounts for most esophageal cancers (>90%) in Taiwan [2]. In our study, we enrolled only squamous cell carcinoma. The standard treatment for nonmetastatic esophageal cancer comprises neoadjuvant CCRT followed by surgery [3, 4]. Only 30%–40% of patients can receive surgery [5]; the local recurrence rate is approximately 46% [8]. Chen et al. studied 298 patients with esophageal cancer and compared the clinical outcomes of neoadjuvant CCRT followed by esophagectomy and CCRT without surgery available in Taiwan Cancer Registry. The HR for death was .56 when surgery was compared with CCRT. Neoadjuvant CCRT followed by esophagectomy was associated with improved OS for locally advanced esophageal squamous cell carcinoma. Therefore, the results revealed the importance of esophagectomy. In both our univariate and multivariate analyses, although there were only 36.2% patients who received operation, surgery was independently associated with better prognosis. Because other factors about treatment, including dose of radiotherapy, days from diagnosis to initial or complete treatment, were not associated with prognosis, if surgical intervention is suitable for a patient with esophageal cancer, esophagectomy should be suggested after CCRT.

Several biomarkers can predict prognosis, including the p53 genotype and miR-200c [24, 25]. However, no biomarker can have a wide application because of complex methodology and high price involved. In this study, we used pre- and post-CCRT platelet counts, NLR, and PLR for predicting esophageal cancer prognosis. All required data were available for almost all patients during treatment.

Thrombocytosis is seen in many patients with cancer [26, 27]. High platelet count is related to poor prognosis in various cancers [28, 29]. Thymidine phosphorylase is a platelet-derived endothelial cell growth factor with potent angiogenic activity [30]. Increase in thymidine phosphorylase levels—expressed at higher levels in solid tumors than in normal tissues [31]—may be associated with poor prognosis in various solid tumor tissues. The importance of high platelet count in esophageal cancer has also been investigated [32]. Shimada et al. reported 374 patients with primary esophageal squamous cell carcinoma. Under a cutoff level of 293 000/*μ*L of platelet count, their multivariate analysis showed that thrombocytosis was an independent prognostic factor (*P* = .009) [33]. Verma et al. showed that thrombocytosis and increased C-reactive protein levels predicted esophageal carcinoma in an advanced stage, with a platelet count of 319,000/*μ*L used as the cutoff level for thrombocytosis [34]. Because cancer-associated mortality is frequently caused by metastasis, recent studies have shown that platelets contribute to all hematogenous tumor extravasation and dissemination steps [35]. In addition to OS and TPD, we try to analyze TM. Here, a high platelet count of 300,000/*μ*L was used as the cutoff level. Before CCRT, 34 patients had high platelet counts (>300,000/*μ*L). The patients with high platelet counts had poor prognosis for OS, TPD, and TM. No study has evaluated prognosis associated with posttreatment platelet counts. We also analyzed the platelet counts after CCRT. In both univariate and multivariate analyses, patients with high platelet counts after treatment had poor prognosis for OS, TPD, and TM. We created 3 groups based on pretreatment and posttreatment platelet counts. Higher platelet counts, whether pre- or post-CCRT, led to poor prognosis. Our results demonstrated that if patients have a high pretreatment or posttreatment platelet count, they may need more aggressive treatment in the future.

Because lymphocytes are critical in promoting systemic immune responses against tumors, lymphocytopenia is associated with poor outcomes in many cancers [36–38]. Fang et al. reported 313 patients with esophageal cancer (stages I–IVA) who received neoadjuvant CCRT. A high ALC during CCRT was associated with a high rate of pathologically complete remission for patients with esophageal cancer [39]. Lymphocytes are sensitive to radiation [40]. The radiotherapy for esophageal cancer is performed for at least 5 weeks, during which time circulating lymphocytes are exposed to a considerable dose of radiation, which can cause lymphocytopenia [39]. Cytotoxic T lymphocytes elicit active and adaptive cellular immunity against tumor cells [41]. In our study, patients with lymphocyte percentage of >16% had better prognosis; however, only lymphocyte percentage, not ALC, was associated with esophageal cancer prognosis.

Inflammatory response biomarkers for esophageal squamous cell carcinoma, including PLR and lymphocyte-to-monocyte ratio, have also been studied. Zhao et al. reported a meta-analysis on the prognostic role of PLR in esophageal cancer: in a total of 6699 patients from 16 studies (17 cohorts), elevated PLR predicted poorer OS (HR, 1.389) and shorter disease-free survival (HR, 1.404) [42]. In their study comprising 60 patients, McLaren et al. reported that NLR and PLR predict treatment responses to neoadjuvant therapy in esophageal cancer. An elevated PLR predicted shorter OS [43]. In a meta-analysis, Yodying et al. reported that both high NLR and PLR significantly predicted poorer OS in 1540 patients [44]. Systemic inflammation is crucial during all tumorigenesis stages. As per a previous study, inflammation may contribute to tumor initiation through genetic mutations, genomic instability, and epigenetic modifications. Inflammation activates tissue repair responses that may induce proliferation and enhance survival of premalignant cells. Inflammation also stimulates angiogenesis, causes immunosuppression, and promotes formation of microenvironments in which malignant cells can survive, ultimately promoting metastatic spread [45]. The close association between increased systemic inflammatory responses, including NLR and PLR, and poor prognosis identified in our study may be associated with inflammatory process activation in cancer cells. However, in our multivariate analysis, no significant association was noted between the inflammatory biomarkers and prognosis. For further confirmation of our results, a larger sample size may be required.

## 5. Conclusions

Poor prognosis with shorter OS, TPD, and TM were noted in nonmetastatic esophageal cancer patients with pre- and post-CCRT high platelet counts (>300,000/*μ*L), particularly at after CCRT, platelet-to-lymphocyte ratio, and neutrophil-to-lymphocyte ratio as well as low lymphocyte percentage. Moreover, surgery remained an independent factor associated with better prognosis. For patients with poor prognosis, operation or more aggressive chemotherapy may be suggested. Our findings may have clinical significance with regard to therapeutic decision-making.

## Figures and Tables

**Table 1:** Clinicopathological characteristics of enrolled patients (n = 105).

	Number (Percentage)	Mean ± Standard deviation

Age	Range: 38~81 y/o	57.69 ± 8.6

Gender:		
Male	98 (93.3%)	
Female	7 (7.6%)	

Pathology		
Squamous cell carcinoma	105 (100%)	

Tumor site		
Upper	28 (26.7%)	
Middle	48 (45.7%)	
Lower	29 (27.6%)	

Stage		
I	13 (12.4%)	
II	27 (25.7%)	
III	65 (61.9%)	

Lab data at diagnosis		
White blood count (/*μ*L)		7940 ± 2616.9
Hb (g/dL)		13.2 ± 1.77
Platelet count (X10^3^/*μ*L)		262.82 ± 110.73
Mean platelet volume (fl)		9.84 ± 0.83
Neutrophil (%)		66.5 ± 11.1
Lymphocyte (%)		23.3 ± 9.5
Monocyte (%)		6.9 ± 2.6

Lab data at finished CCRT		
White blood count (/*μ*L)		3845.7 ± 1832.8
Hb (g/dL)		10.9 ± 1.6
Platelet count (X10^3^/*μ*L)		174.88 ± 81.1
Mean platelet volume (fl)		9.35 ± 0.92
Neutrophil (%)		79.7 ±11.2
Lymphocyte (%)		9.4 ± 7.9
Monocyte (%)		8.5 ± 5

Chemotherapy cycles		
2 cycles	96 (91.4%)	
1 cycle	9 (8.6%)	

Radiotherapy	105 (100%)	
Dose 4000 ~ 5000cGy	40 (38.1%)	
Dose > 5000cGy	65 (61.9%)	

Post-CCRT response		
Complete response	38 (36.2%)	
Partial response	48 (45.7%)	
Stable disease	2 (1.9%)	
Progressive disease	17 (16.2%)	
Overall response	86 (81.9%)	
Clinical benefit	88 (83.8%)	

Post-CCRT treatment		
Operation	38 (36.2%)	
Chemotherapy	45 (42.9%)	
Non-treatment	22 (20.9%)	

Expired	56 (53.3%)	
Overall survival (days)	(92~1844 days)	586 ± 423.5

Progressive rate	62 (59%)	
Time to progressive disease (days)		414.6 ± 355.5

Metastatic rate	56 (53.3%)	
Time to metastasis (days)		425.8 ± 356.8

**Table 2:** Univariate and multivariate analyses of association between clinical parameters and overall survival.

Clinical features	Patient numbers	Univariate		Multivariate	
		HR^†^ (95% CI^‡^)	P-value	HR^†^ (95% CI^‡^)	P-value

Stage (I vs II vs III)	13 : 27 : 65	-	**0.001** ^∗^	1.907 (1.147-3.171)	**0.013** ^∗^

Tumor site (Upper vs Middle vs Lower)	28 : 48 : 29	-	0.905	-	-

Gender (Male vs Female)	98 : 7	0.737 (0.248-2.009)	0.513	-	-

Age (≥ vs <60 years old)	40 : 65	0.726 (0.399-1.260)	0.241	-	-

Surgery (Yes vs No)	38 : 67	2.046 (1.154-3.334)	**0.013** ^∗^	0.353 (0.182-0.687)	**0.002** ^∗^

Radiotherapy (4000~5000cGy vs >5000cGy)	40 : 65	0.7847 (0.462-1.344)	0.381	-	-

Days from diagnosis to initial treatment (< v.s ≥29)	57 : 48	0.989 (0.583-1.677)	0.966	-	-

Days from diagnosis to complete treatment (< v.s ≥69)	54 : 51	0.704 (0.405-1.185)	0.180	-	-

Hematologic Markers					

Platelet count of pre-treatment (≥ vs <300,000/*μ*L)	34 : 71	0.535 (0.277-0.887)	**0.018** ^∗^	1.543 (0.778-3.061)	0.217

Platelet count of post-treatment (≥ vs <300,000/*μ*L)	9 : 96	0.389 (0.078-0.704)	**0.010** ^∗^	2.656 (1.047-6.735)	**0.041** ^∗^

MPV^§^ (≥ vs <9.8fl)	51 : 50^&^	1.286 (0.750-2.207)	0.361	-	-

Hemoglobin (≥ vs <14g/dL)	17 : 88	1.426 (0.745-2.583)	0.302	-	-

White blood count (≥ vs <10,000/*μ*L)	12 : 93	0.715 (0.255-1.795)	0.432	-	-

Absolute Neutrophil count (≥ vs <4483/*μ*L)	53 : 52	0.617 (0.362-1.041)	0.070	-	-

Neutrophil percentage (≥ vs <73.4%)	53 : 52	0.729 (0.431-1.232)	0.238	-	-

Absolute Monocyte count (≥ vs <449/*μ*L)	53 : 52	0.756 (0.444-1.276)	0.292	-	-

Monocyte percentage (≥ vs <7%)	53 : 52	0.955 (0.564-1.615)	0.861	-	-

Absolute lymphocyte counts (≥ vs <1042/*μ*L)	53 : 52	1.627 (0.969-2.823)	0.065	-	-

Lymphocyte percentage (≥ vs <16%)	53 : 52	1.997 (1.205-3.548)	**0.008** ^∗^	0.431 (0.131-1.419)	0.168

Biomarker of Inflammation					

Platelet to-ALC^∧^ ratio (≥ vs <236)	53 : 52	0.632 (0.368-1.064)	0.083	-	-

Platelet-to-Lymphocyte(%) ratio (≥ vs <14605)	53 : 52	0.514 (0.316-0.911)	**0.021** ^∗^	0.542 (0.245-1.200)	0.133

Neutrophil-to-Lymphocyte ratio (≥ vs <4.35)	53 : 52	0.507 (0.290-0.847)	**0.010** ^∗^	0.804 (0.258-2.504)	0.709

^†^HR, hazard ratio; ^‡^CI, confidence interval; ^§^MPV, mean platelet volume; ^∧^ALC, absolute lymphocyte count; ^&^no data of 4 patients.

**Table 3:** Univariate and multivariate analyses of association between clinical parameters and time to progressive disease.

Clinical features	Patient numbers	Univariate		Multivariate	
		HR^†^ (95% CI^‡^)	P-value	HR^†^ (95% CI^‡^)	P-value

Stage (I vs II vs III)	13 : 27 : 65	-	**0.008** ^∗^	1.668 (1.040-2.676)	**0.035** ^∗^

Tumor site (Upper vs Middle vs Lower)	28 : 48 : 29	-	0.514	-	-

Gender (Male vs Female)	98 : 7	0.388 (0.069-0.736)	**0.014** ^∗^	2.184 (0.947-5.040)	0.068

Age (≥ vs <60 years old)	40 : 65	0.766 (0.434-1.303)	0.309	-	-

Surgery (Yes vs No)	38 : 67	1.850 (1.090-2.996)	**0.022** ^∗^	0.413 (0.225-0.760)	**0.005** ^∗^

Radiotherapy (4000~5000cGy vs >5000cGy)	40 : 65	0.779 (0.469-1.301)	0.348	-	-

Days from diagnosis to initial treatment (< v.s ≥29)	57 : 48	1.251 (0.758-2.062)	0.382	-	-

Days from diagnosis to complete treatment (< v.s ≥69)	54 : 51	0.821 (0.493-1.354)	0.433	-	-

Hematologic Markers					

Platelet count of pre-treatment (≥ vs <300,000/*μ*L)	34 : 71	0.580 (0.303-0.956)	**0.035** ^∗^	1.326 (0.681-2.582)	0.409

Platelet count of post-treatment (≥ vs <300,000/*μ*L)	9 : 96	0.456 (0.105-0.960)	**0.042** ^∗^	2.538 (1.024-6.291)	**0.045** ^∗^

MPV^§^ (≥ vs <9.8fl)	51 : 50^&^	0.849 (0.508-1.411)	0.522	-	-

Hemoglobin (≥ vs <14g/dL)	17 : 88	1.402 (0.754-2.477)	0.303	-	-

White blood count (≥ vs <10,000/*μ*L)	12 : 93	0.648 (0.230-1.516)	0.274	-	-

Absolute Neutrophil count (≥ vs <4483/*μ*L)	53 : 52	0.740 (0.444-1.218)	0.232	-	-

Neutrophil percentage (≥ vs <73.4%)	53 : 52	0.809 (0.490-1.332)	0.403	-	-

Absolute Monocyte count (≥ vs <449/*μ*L)	53 : 52	0.961 (0.583-1.583)	0.874	-	-

Monocyte percentage (≥ vs <7%)	53 : 52	0.803 (0.486-1.321)	0.385	-	-

Absolute lymphocyte counts (≥ vs <1042/*μ*L)	53 : 52	1.615 (0.987-2.725)	0.056	-	-

Lymphocyte percentage (≥ vs <16%)	53 : 52	1.838 (1.134-3.160)	**0.015** ^∗^	0.336 (0.053-2.131)	0.250

Biomarker of Inflammation					

Platelet to-ALC^∧^ ratio (≥ vs <236)	53 : 52	0.535 (0.309-0.865)	**0.012** ^∗^	0.854 (0.399-1.826)	0.685

Platelet-to-Lymphocyte(%) ratio (≥ vs <14605)	53 : 52	0.511 (0.296-0.826)	**0.007** ^∗^	0.971 (0.412-2.291)	0.947

Neutrophil-to-Lymphocyte ratio (≥ vs <4.35)	53 : 52	0.571 (0.339-0.933)	**0.026** ^∗^	0.453 (0.077-2.678)	0.385

^†^HR, hazard ratio; ^‡^CI, confidence interval; ^§^MPV, mean platelet volume; ^∧^ALC, absolute lymphocyte count; ^&^no data of 4 patients.

**Table 4:** Univariate and multivariate analyses of association between clinical parameters and time to metastasis.

Clinical features	Patient numbers	Univariate		Multivariate	
		HR^†^ (95% CI^‡^)	P-value	HR^†^ (95% CI^‡^)	P-value

Stage (I vs II vs III)	13 : 27 : 65	-	**0.004** ^∗^	1.732 (1.029-2.915)	**0.040** ^∗^

Tumor site (Upper vs Middle vs Lower)	28 : 48 : 29	-	0.430	-	-

Gender (Male vs Female)	98 : 7	0.400 (0.068-0.850)	**0.027** ^∗^	2.146 (0.876-5.258)	0.097

Age (≥ vs <60 years old)	40 : 65	0.759 (0.417-1.327)	0.316	-	-

Surgery (Yes vs No)	38 : 67	1.814 (1.039-3.013)	**0.036** ^∗^	0.441 (0.232-0.839)	**0.013** ^∗^

Radiotherapy (4000~5000cGy vs >5000cGy)	40 : 65	0.775 (0.455-1.334)	0.363	-	-

Days from diagnosis to initial treatment (< v.s ≥29)	57 : 48	1.161 (0.686-1.966)	0.578	-	-

Days from diagnosis to complete treatment (< v.s ≥69)	54 : 51	0.789 (0.460-1.334)	0.369	-	-

Hematologic Markers					

Platelet count of pre-treatment (≥ vs <300,000/*μ*L)	34 : 71	0.510 (0.247-0.838)	**0.012** ^∗^	1.282 (0.642-2.560)	0.484

Platelet count of post-treatment (≥ vs <300,000/*μ*L)	9 : 96	0.372 (0.060-0.681)	**0.010** ^∗^	2.926 (1.164-7.355)	**0.023** ^∗^

MPV^§^ (≥ vs <9.8fl)	51 : 50^&^	0.864 (0.504-1.476)	0.588	-	-

Hemoglobin (≥ vs <14g/dL)	17 : 88	1.502 (0.785-2.688)	0.235	-	-

White blood count (≥ vs <10,000/*μ*L)	12 : 93	0.556 (0.171-1.281)	0.139	-	-

Absolute Neutrophil count (≥ vs <4483/*μ*L)	53 : 52	0.698 (0.408-1.177)	0.175	-	-

Neutrophil percentage (≥ vs <73.4%)	53 : 52	0.808 (0.476-1.365)	0.423	-	-

Absolute Monocyte count (≥ vs <449/*μ*L)	53 : 52	1.000 (0.591-1.692)	0.999	-	-

Monocyte percentage (≥ vs <7%)	53 : 52	0.973 (0.574-1.647)	0.917	-	-

Absolute lymphocyte counts (≥ vs <1042/*μ*L)	53 : 52	1.628 (0.968-2.819)	0.065	-	-

Lymphocyte percentage (≥ vs <16%)	53 : 52	1.834 (1.100-3.225)	**0.021** ^∗^	0.437 (0.065-2.943)	0.398

Biomarker of Inflammation					

Platelet to-ALC^∧^ ratio (≥ vs <236)	53 : 52	0.455 (0.254-0.750)	**0.003** ^∗^	0.955 (0.420-2.174)	0.913

Platelet-to-Lymphocyte(%) ratio (≥ vs <14605)	53 : 52	0.439 (0.248-0.727)	**0.002** ^∗^	1.129 (0.443-2.879)	0.801

Neutrophil-to-Lymphocyte ratio (≥ vs <4.35)	53 : 52	0.569 (0.330-0.955)	**0.033** ^∗^	0.506 (0.082-3.130)	0.466

^†^HR, hazard ratio; ^‡^CI, confidence interval; ^§^MPV, mean platelet volume; ^∧^ALC, absolute lymphocyte count; ^&^no data of 4 patients.

## Data Availability

The datasets generated during and analyzed during the current study are not publicly available due to patient privacy but are available from the corresponding author upon reasonable request.
